# Early Prediction Model for Critical Illness of Hospitalized COVID-19 Patients Based on Machine Learning Techniques

**DOI:** 10.3389/fpubh.2022.880999

**Published:** 2022-05-24

**Authors:** Yacheng Fu, Weijun Zhong, Tao Liu, Jianmin Li, Kui Xiao, Xinhua Ma, Lihua Xie, Junyi Jiang, Honghao Zhou, Rong Liu, Wei Zhang

**Affiliations:** ^1^Department of Clinical Pharmacology, Xiangya Hospital, Central South University, Changsha, China; ^2^National Clinical Research Center for Geriatric Disorders, Changsha, China; ^3^Shenzhen Center for Chronic Disease Control, Shenzhen, China; ^4^Department of Pulmonary and Critical Care Medicine, Hunan Provincial People's Hospital, The First Affiliated Hospital of Hunan Normal University, Changsha, China; ^5^Department of Pulmonary and Critical Care Medicine, The Second Xiangya Hospital, Central South University, Changsha, China; ^6^Union Hospital, Tongji Medical College, Huazhong University of Science and Technology, Wuhan, China; ^7^B7 Department, Zhongfa District of Tongji Hospital, Tongji Medical, Huazhong University of Science and Technology, Wuhan, China

**Keywords:** COVID-19, risk factors, critical illness, machine learning, LASSO regression

## Abstract

**Motivation:**

Patients with novel coronavirus disease 2019 (COVID-19) worsen into critical illness suddenly is a matter of great concern. Early identification and effective triaging of patients with a high risk of developing critical illness COVID-19 upon admission can aid in improving patient care, increasing the cure rate, and mitigating the burden on the medical care system. This study proposed and extended classical least absolute shrinkage and selection operator (LASSO) logistic regression to objectively identify clinical determination and risk factors for the early identification of patients at high risk of progression to critical illness at the time of hospital admission.

**Methods:**

In this retrospective multicenter study, data of 1,929 patients with COVID-19 were assessed. The association between laboratory characteristics measured at admission and critical illness was screened with logistic regression. LASSO logistic regression was utilized to construct predictive models for estimating the risk that a patient with COVID-19 will develop a critical illness.

**Results:**

The development cohort consisted of 1,363 patients with COVID-19 with 133 (9.7%) patients developing the critical illness. Univariate logistic regression analysis revealed 28 variables were prognosis factors for critical illness COVID-19 (*p* < 0.05). Elevated CK-MB, neutrophils, PCT, α-HBDH, D-dimer, LDH, glucose, PT, APTT, RDW (SD and CV), fibrinogen, and AST were predictors for the early identification of patients at high risk of progression to critical illness. Lymphopenia, a low rate of basophils, eosinophils, thrombopenia, red blood cell, hematocrit, hemoglobin concentration, blood platelet count, and decreased levels of K, Na, albumin, albumin to globulin ratio, and uric acid were clinical determinations associated with the development of critical illness at the time of hospital admission. The risk score accurately predicted critical illness in the development cohort [area under the curve (AUC) = 0.83, 95% CI: 0.78–0.86], also in the external validation cohort (*n* = 566, AUC = 0.84).

**Conclusion:**

A risk prediction model based on laboratory findings of patients with COVID-19 was developed for the early identification of patients at high risk of progression to critical illness. This cohort study identified 28 indicators associated with critical illness of patients with COVID-19. The risk model might contribute to the treatment of critical illness disease as early as possible and allow for optimized use of medical resources.

## Introduction

The coronavirus disease 2019 (COVID-19) pandemic is spreading worldwide. As a communicable disease, COVID-19 is caused by severe acute respiratory syndrome coronavirus 2. Until 14 February 2022, the WHO reported 412,044,520 COVID-19 confirmed cases globally, with an average mortality rate of 1.4%. The clinical spectrum of COVID-19 infection ranges from asymptomatic infection, and mild upper respiratory tract illness to critically ill cases (1). It has been reported that about 5% of patients with COVID-19 infection experience rapid deterioration from the onset of symptoms into critical illness (2) and with a mortality rate of 61.5% for critical ones within 28 days of hospital admission (3). Treatment of patients with critical illnesses constitutes great pressure on medical services, especially results in the lack of intensive care resources. Therefore, early identification and effective triaging of patients with a high risk of developing critical illness COVID-19 upon admission can aid in improving patient care, increasing the cure rate, and mitigating the burden on the medical care system.

The risk factors for critical illness are not well-revealed. Previous reports have identified that older age, organ dysfunction, neutrophilia, preexisting concurrent cardiovascular or cerebrovascular diseases, coagulopathy, amounts of CD3+CD8+ T cells, and elevated D-dimer levels are associated with the development of acute respiratory distress syndrome and increased mortality risk (1, 4–9). A limited number of publications have identified chest radiographic abnormality, older age, hemoptysis, dyspnea, unconsciousness, number of comorbidities, cancer history, neutrophil-to-lymphocyte ratio (10), lactate dehydrogenase (LDH), and direct bilirubin are risk factors associated with the development of critical illness (11, 12). Clinical scores for predicting which patients with COVID-19 will develop critical illness were developed with these above 10 factors (11, 12), which show well-discrimination. In addition, an integrated model was developed with patient history, laboratory markers, and chest radiography at hospital admission to predict critical illness by Schalekamp et al. (13). However, in these models, some diagnoses of co-existing illness and symptoms were from patients' self-reports at admission, which might lead to recall bias.

Mathematical modeling with appropriate inputs can make predictions in the dynamics and control of the infectious disease. A series of mathematical models have been developed on the transmission dynamics and control of COVID-19 or SARS-CoV-2 virus in different countries (14–24), namely, Wuhan, Italy, and the USA. In this retrospective multicenter study, we proposed and extended classical least absolute shrinkage and selection operator (LASSO) logistic regression for the early identification of patients at high risk of progression to critical illness. We systematically analyzed the accessible laboratory findings of confirmed 1,929 patients with COVID-19 having clear prognostic information in 32 hospitals in Hubei and Hunan provinces of China and identified robust and meaningful factors associated with a critical illness. The laboratory findings were measured objectively. A risk prediction model was constructed according to LASSO logistic regression to help identify patients at the time of hospital admission who are at high risk of developing a critical illness. This model aims at distinguishing patients at imminent risk of critical illness, thereby optimizing the allocation of limited healthcare resources and potentially lowering the mortality rate.

## Methods

### Data Collection

This study has been proved by the Institute of Clinical Pharmacology, Central South University. For the urgent need to collect and analyze data on this emerging pathogen, the ethics committee of the Institute of Clinical Pharmacology, Central South University granted a waiver of written informed consent from study participants. Medical records of hospitalized patients with COVID-19 diagnosed in 31 hospitals in China (4 hospitals in Hubei Province and 27 hospitals in Hunan Province) were collected. All patients who were diagnosed with COVID-19 by positive high-throughput sequencing or real-time reverse-transcription PCR (RT-PCR) assay for nasal and pharyngeal swab specimens were screened, our study enrolled all adult inpatients (≥18 years old) who were hospitalized for COVID-19 and had an explicit outcome of critical illness. The data were cross-checked by experienced respiratory clinicians. All patients with data on clinical status at hospitalization (laboratory findings, critical illness, and discharge status) were included.

### Clinical Outcome

The outcome of this study is a critical illness, which is defined as a composite of invasive ventilation, admission to the intensive care unit (ICU), or fatal of patients with COVID-19 (25). The follow-up time was calculated from the first day of hospitalization to the date of death or discharge, or the censored date (12th April 2020 for the development cohort and 11 June 2020 for the validation cohort).

### Potential Predictive Variables

Demographic variables and laboratory findings of patients at hospital admission were collected as potential predictive variables. Demographic variables included age and gender. Laboratory findings were conducted as the first measurement within 2 days after at admission, laboratory indexes with complete measurements for more than 50% of the patients in the development cohort were collected: hematologic (hematokrit, basophils, eosinophils, lymphocytes, monocytes, neutrophils, mean corpuscular volume, hemoglobin concentration, coefficient of variation [CV] and SD of red blood cell volume distribution width [RDW], blood platelet count, thrombocytocrit, red blood cell, and white blood cells), biochemical [levels of glucose, K, Na, total Ca, Cl, total protein, lactate dehydrogenase (LDH), glutamic-pyruvic transaminase, creatine kinase, aspartate transaminase (AST), creatine kinase muscle-brain isoform (CK-MB), creatinine, ureophil, albumin, globulin, albumin to globulin ratio, and glomerular filtration rate (GFR)], coagulation function indexes [levels of D-dimer and fibrinogen, activated partial thromboplastin time (APTT), and prothrombin time (PT)], infection-related indices [levels of C-reactive protein (CRP), procalcitonin (PCT), and alpha hydroxybutyrate dehydrogenase (α-HBDH)], and also the level of uric acid. For the complete laboratory findings and corresponding ratio of missing values, please refer to Supplementary Table 1.

### Statistical Analysis

Continuous and categorical variables were presented as mean, SD [interquartile range (IQR)], and *n* (%), respectively.

A total of 1,255 patients hospitalized with COVID-19 in the development cohort were included for variable selection. To access the association between the quantitative laboratory findings described above and the occurrence of critical illness, a univariate logistic regression analysis was conducted. Since the odds ratio (OR) is interpreted per unit change, to standardize ORs between variables with a different range, logistic regression analysis was applied to dichotomies data (1 = with the occurrence of critical illness and 0 = without the occurrence of critical illness) with quartiles of each of the 38 laboratory findings modeled as continuous (<25% quartile = 1; ≥25% and <50% quartile = 2; ≥50% quartile and <75% quartile = 3; and ≥ 75% quartile = 4). The associations between the occurrence of critical illness and age (≥55 vs. <55 years) were also evaluated.

The statistically significant 28 covariates (*p* < 0.05) in the univariate logistic analysis were selected as candidates for risk score development of critical illness. A total of 1,064 patients with at least 80% data completeness of the above 28 variables were utilized for model establishment. We applied predictive mean matching to impute numeric features (laboratory findings) with “mice” packages in R for these 1,064 patients.

Prediction models were developed with the LASSO logistic regression, support vector regression (SVR), artificial neural network (ANN), regression tree (RT), and multivariate adaptive regression splines (MARS) machine learning techniques. We used the “glmnet” (14) package for LASSO, “e1071” package for SVR, “RSNNS” package for ANN, “rpart” package for RT, and “earth” package for MARS. Default parameters were used. L1-penalized least absolute shrinkage and selection regression augmented with 1,000-fold cross-validation for internal validation was utilized. LASSO logistic regression is a logistic regression model that penalizes the absolute size of the coefficients of a regression model according to the value of λ. In the process of LASSO regression coefficients, some unimportant regression coefficients can be directly reduced to 0 to achieve the function of variable screening. In comparison to the ridge regression model, the penalty term in the LASSO regression is an absolute value, namely, L1 regular. The estimates of weaker factors shrink toward zero with larger penalties, then only the greatest predictors were left in the model. We select the most predictive covariates by the minimum value of λ. Subsequently, variables identified by LASSO regression analysis were used to construct the risk score with their coefficients:


(1) $$Risk\;Score(RS)=\sum_{i=1}^{n}\left(Value_{i}\;*\;Coe_{i}\right)$$

where *n* stands for the number of prognostic variables in the model; Value_i_ is the original value of variable_i_; and Coe_i_ is the estimated coefficient of Value_i_ in the LASSO logistic regression model. The probability of developing critical illness was calculated with the following formula: probability = exp (RS)/[1+ exp(RS)].

We used receiver operating characteristic (ROC) curves to compare the sensitivity and specificity of scores generated with different machine learning techniques. The abscissa and ordinate coordinates of ROC curves are false-positive rate and true probability, respectively. The points of ROC curves reflect the susceptibility to the same signal stimulus. By comparing the false-positive and true numbers, ROC curves show the performance of a classification model at all classification thresholds. The area under the receiver operating characteristics (AUROC), namely, the entire two-dimensional area underneath the entire ROC curve, was used as the precision measurement. AUROC shows how much the model is capable of distinguishing between classes. The larger the AUROC value, the better will be the model at predicting different classes. R-package “ROCR” was utilized for the calculation of the AUROC curve.

To explore temporal changes in laboratory findings during hospitalization, differences between critical illness groups during follow-up in laboratory findings were estimated from linear mixed models with R package “nlme.”

**Figure 1:**
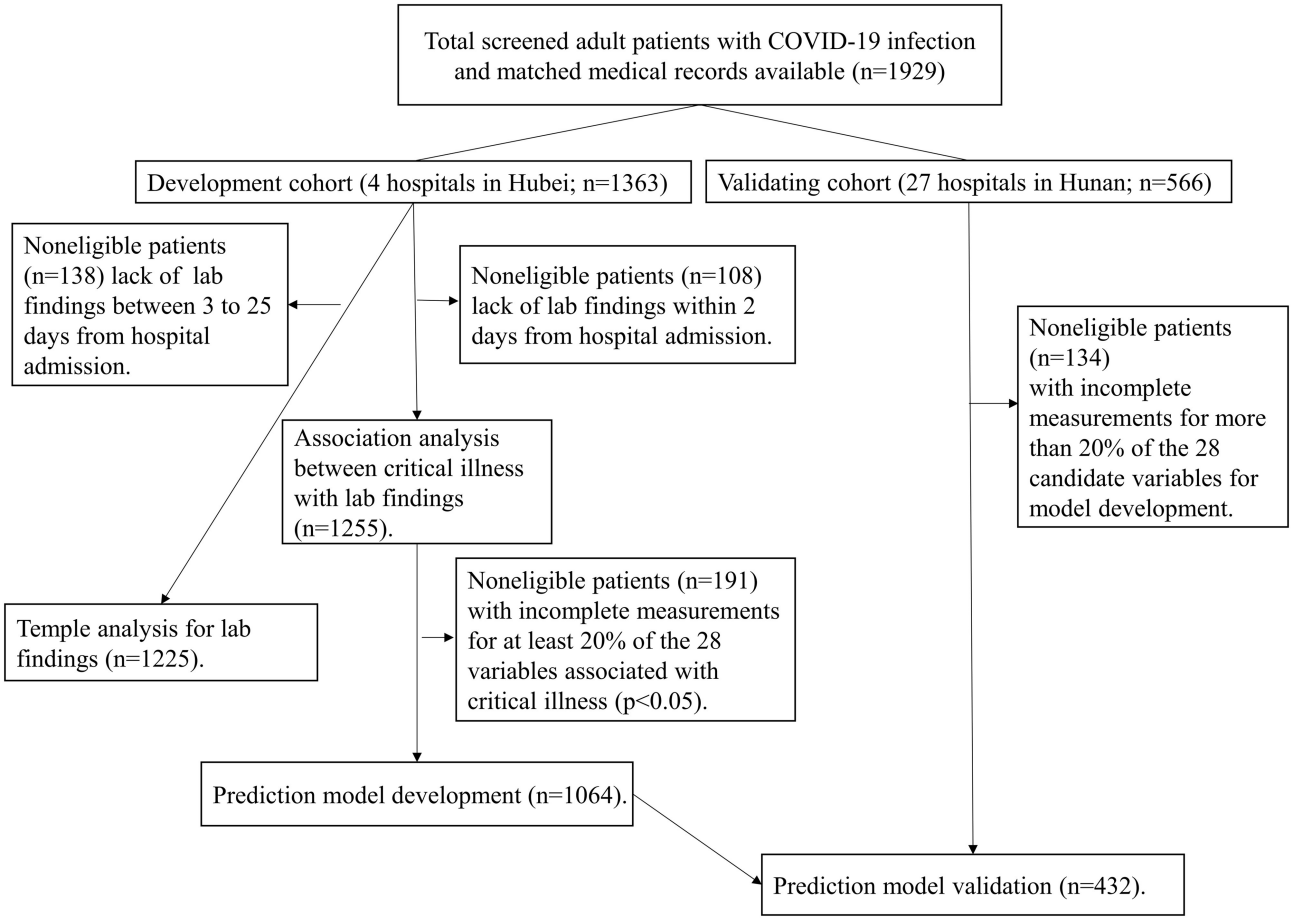
Study flowchart detailing which samples were utilized at each phase of statistical analysis. COVID-19: severe coronavirus disease 2019.

Details of samples used at each stage of statistical analysis were depicted in Figure 1. All statistical analysis was conducted with R software (version 3.6.2, R Foundation), and *p*-values were computed from two-tailed tests of statistical significance with a type I error rate of 5%.

### External Model Validation

To validate the generalizability of the risk scores, we used an independent cohort from hospitals in Hunan province including 566 patients. We collected the same variables required for calculating the risk score from the validation cohort and cross-checked them. The 432 patients with at least 80% data completeness of the 28 variables used for model development were selected. The laboratory findings were imputed and the risk score was calculated as described for the development cohort. To assess the discriminative ability, the AUCs were evaluated.

## Results

### Characteristics of the Cohorts

The development cohort with 1,363 patients, of which a total of 133 patients eventually developed critical illness (9.8%), from 4 hospitals in Hubei. The median follow-up time for patients was 14 days. The average (SD) age of patients in this cohort was 57.84 (16.29) years; 634 patients (46.52%) were men. The validation cohort included 566 patients with a mean (SD) age of 45.94 (15.33) years, 291 (51.41) were men. The median follow-up time for patients was 13 days. The critical illness eventually developed in 28 (4.24%) of these patients.

### Prognostic Factors of Critical Illness

**Figure 2:**
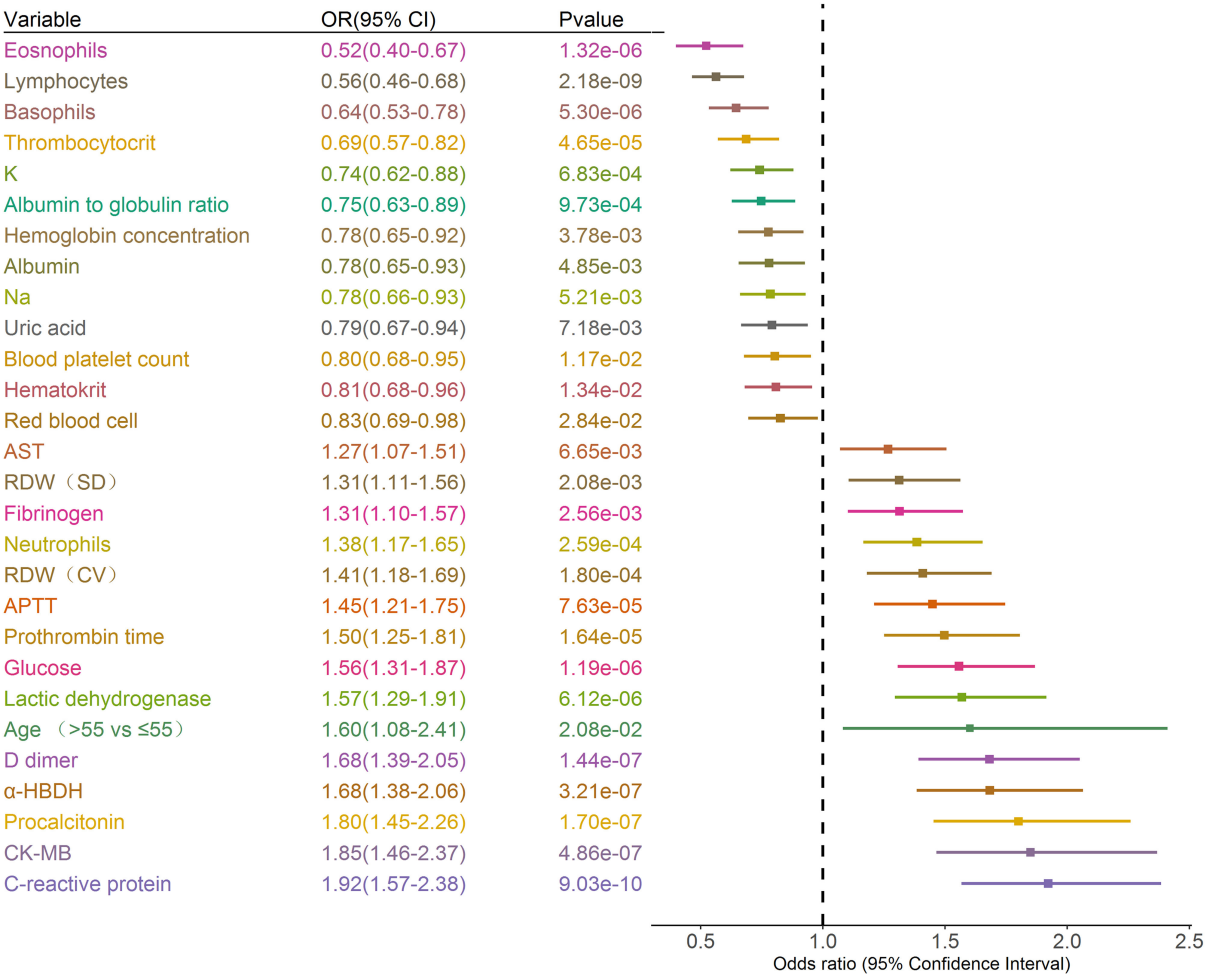
Prognostic associations of clinical characteristics and laboratory findings in the development dataset. Unadjusted ORs (boxes) and corresponding 95% CIs (horizontal lines) for variables associated with the development of critical illness are represented. Box size is inversely proportional to the standard error of OR. The variables are stratified as quartiles. OR, odds ratio. CI, confidence interval.

A total of 39 features were tested for associations with critical illness in the development cohort with univariate logistic regression analysis. The results of the 1,255 patients showed that 28 variables were prognosis factors for critical illness COVID-19 (*p* < 0.05, Table 1, Figure 2). The odds of critical illness were higher in patients older than 65 years. Laboratory results show that elevated CK-MB, neutrophils, PCT, α-HBDH, D-dimer, LDH, glucose, PT, APTT, RDW (SD and CV), fibrinogen, and AST were associated with a critical illness. Patients in the critical illness group showed lymphopenia and had a low rate of basophils, eosinophils, thrombopenia, red blood cell, hematocrit, hemoglobin concentration, and blood platelet count and represented decreased levels of K, Na, albumin, albumin to globulin ratio, and uric acid, compared with the non-critical illness group.

**Table 1:** Laboratory characteristics among patients who did not or did develop critical illness in the development cohort.

**Laboratory tests**	**Total, mean (SD) [Interquartile range]**	**Critical illness (*n* = 1,255)**	
		**No (*n* = 1,130)**	**Yes (*n* = 125)**
**Hematologic**			
Lymphocytes, ×10^9^/L	1.32 (0.67) [0.86–1.66]	1.36 (0.67) [0.9–1.69]	0.94 (0.47) [0.6–1.23]
Eosnophils, ×10^9^/L	0.08 (0.14) [0–0.1]	0.08 (0.14) [0.01–0.11]	0.05 (0.13) [0–0.05]
Basophils, ×10^9^/L	0.02 (0.02) [0.01–0.03]	0.03 (0.02) [0.01–0.04]	0.02 (0.03) [0.01–0.02]
Neutrophils, ×10^9^/L	4.19 (2.85) [2.54–4.76]	4.03 (2.61) [2.5–4.66]	5.57 (4.24) [2.84–7.97]
Blood platelet, ×10^9^/L	219.31 (84.12) [161–266]	220.91 (83.06) [164.75–267.25]	205.43 (91.92) [135–263.25]
Thrombocytocrit, %	0.22 (0.08) [0.16–0.27]	0.22 (0.08) [0.17–0.27]	0.19 (0.08) [0.14–0.24]
RDW (CV),%	12.91 (1.45) [12–13.3]	12.86 (1.41) [12–13.3]	13.37 (1.75) [12.22–14.03]
RDW (SD), fL	41.59 (4.48) [38.7–43.7]	41.4 (4.21) [38.7–43.6]	43.27 (6.22) [39.8–45.48]
Hematokrit, %	37.74 (6.04) [34.4–41.5]	37.89 (5.97) [34.7–41.62]	36.32 (6.53) [31.4–40.48]
Hemoglobin concentration, g/L	126.56 (18.71) [116–139]	127.17 (18.37) [117–139]	121.15 (20.8) [107–135]
Red blood cells, ×10^12^/L	5.12 (11.5) [3.7–4.63]	5.04 (10.56) [3.72–4.66]	5.79 (17.68) [3.35–4.45]
**Biochemical**			
AST, U/L	29.04 (21.34) [16.7–33.35]	28.51 (21.03) [16.5–32.8]	33.46 (23.4) [18.4–41.5]
CK-MB, U/L	9.51 (9.13) [5–11.4]	9.14 (9.28) [5–10.85]	11.99 (7.56) [7–13.5]
Albumin to globulin ratio, %	1.34 (0.34) [1.11–1.54]	1.35 (0.34) [1.12–1.56]	1.26 (0.34) [1.08–1.44]
Albumin, g/L	37.1 (5.58) [33.4–41.3]	37.27 (5.52) [33.67–41.4]	35.62 (5.92) [31.82–39.5]
LDH, U/L	224.76 (117.43) [153–254]	217.94 (111.64) [151–240]	270.02 (142.8) [173–331.5]
Glucose, mmol/L	6.49 (2.91) [4.93–6.93]	6.37 (2.78) [4.91–6.79]	7.49 (3.66) [5.5–8.09]
K, mmol/L	4.12 (0.54) [3.8–4.44]	4.13 (0.53) [3.8–4.45]	3.98 (0.59) [3.68–4.32]
Na, mmol/L	139.91 (4.29) [137.6–142.7]	140.05 (4.14) [138–142.8]	138.73 (5.34) [135.55–142]
**Infection-related indices**			
CRP, mg/L	28.8 (41.98) [2.4–40.6]	26.09 (38.96) [2.2–38]	53.9 (57.96) [13.25–64.4]
PCT, ng/ml	0.28 (1.7) [0.04–0.09]	0.17 (0.63) [0.04–0.08]	1.04 (4.38) [0.05–0.22]
α-HBDH, U/L	173.23 (85.83) [120–192]	166.55 (79.52) [117–186]	210.97 (108.1) [145.75–261.75]
**Coagulation function**			
D-dimer, μg/mL	2.09 (7.43) [0.26–1.45]	1.8 (6.61) [0.24–1.3]	4.29 (11.79) [0.46–3.12]
PT, s	11.57 (1.09) [10.9–12]	11.49 (0.97) [10.8–12]	12.18 (1.61) [11.2–12.6]
APTT, s	28.12 (6.21) [24.4–30.7]	27.78 (6.04) [24.4–30.3]	30.58 (6.89) [25.5–33.9]
Fibrinogen, g/L	3.18 (1.21) [2.31–3.69]	3.15 (1.23) [2.29–3.64]	3.39 (1.06) [2.65–3.98]
Uric acid, umol/L	283.57 (108.58) [212–332]	284.9 (105.29) [214.25–336]	272.18 (133.43) [200–291.5]

*RDW, red blood cell volume distribution width; AST, aspartate aminotransferase; CV, coefficient of variation; SD, standard deviation; AST, aspartate aminotransferase; CK-MB, Creatine kinase muscle-brain isoform; LDH, lactate dehydrogenase; CRP, C-reactive protein; PCT, Procalcitonin; PT, prothrombin time; APTT, activated partial thromboplastin time*.

### Longitudinal Observations of Laboratory Variables

**Figure 3:**
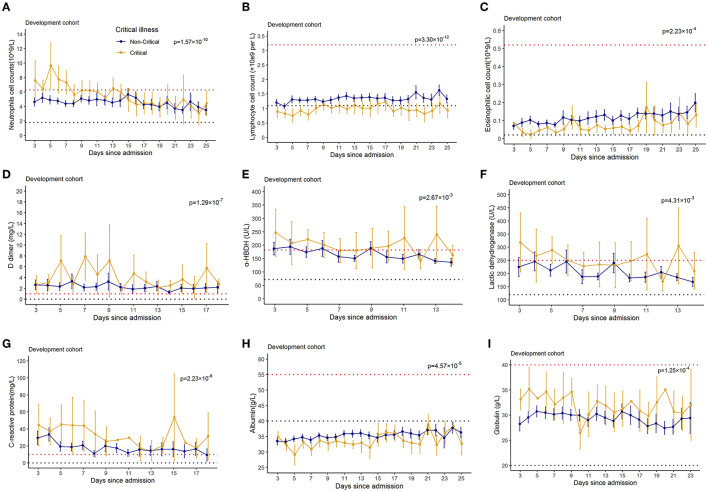
Temporal changes in laboratory findings from illness onset in patients hospitalized with COVID-19. Temporal changes in neutrophils **(A)**, lymphocytes **(B)**, eosinophils **(C)**, D-dimer **(D)**, alpha hydroxybutyrate dehydrogenase **(E)**, lactate dehydrogenase **(F)**, C-reaction protein **(G)**, albumin **(H)**, and glucose **(I)** in the development dataset were presented. Differences between critical illness patients and non-critical illness patients were demonstrated with *p*-values calculated with mixed linear models. The dashed lines in black and red color show the lower and upper normal limits of each laboratory finding.

To determine the major clinical features that appeared during COVID-19 disease progression, the dynamic changes in 28 clinical laboratory parameters were measured within 2 days after hospital admission and associated with critical illness, namely, hematological and biochemical parameters, were recorded from day 3 to day 25 after hospital admission. The temporal changes in laboratory findings during hospitalization were explored (Figure 3). Baseline lymphocyte count was significantly lower in critical illness than in non-critical illness patients. Levels of CRP, D-dimer, LDH, and glucose were clearly elevated in the critical illness group compared with the non-critical illness group throughout the clinical course either in the developing dataset. Furthermore, we found that compared to that in the non-critical illness group, neutrophils, α-HBDH, and globulin were increased in the critical illness group, while eosinophils and albumin were decreased in the critical illness group.

### Construction of the Risk Models and their Performances

**Figure 4:**
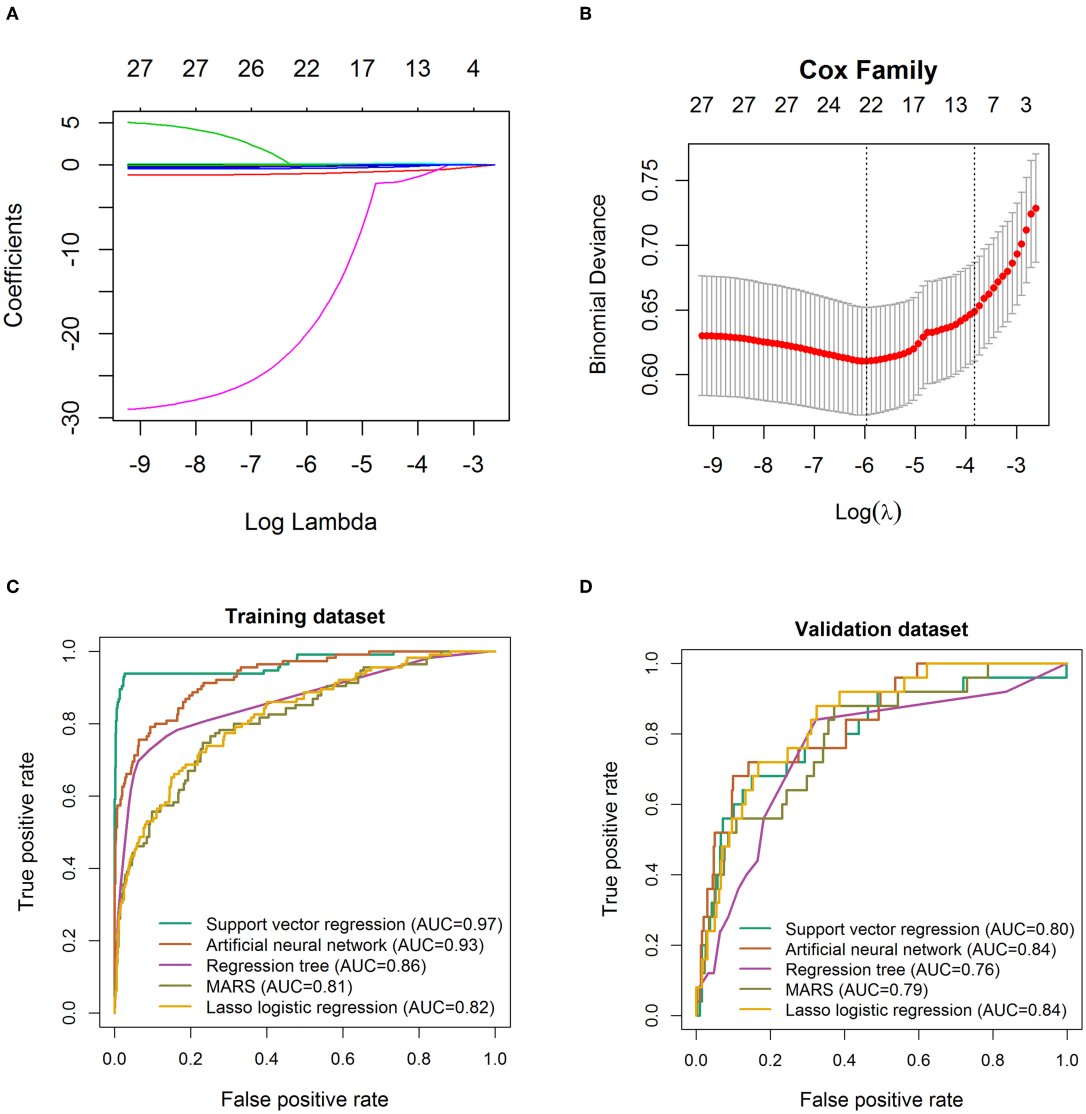
Feature selection using the least absolute shrinkage and selection operator (LASSO) logistic regression model. **(A)** LASSO coefficient profiles of the 29 baseline features. **(B)** Tuning parameter (λ) selection in the LASSO model used 1,000-fold cross-validation *via* minimum criteria. Receiver operating characteristic curve for the performance of different machine learning techniques to distinguish individuals with COVID-19 from those with critical illness COVID-19 in the training cohort **(C)** and validation cohort 1 **(D)**, respectively. AUC, area under the receiver operating characteristic curve. The true positive rate represents module sensitivity, whereas the false positive rate is one minus the specificity.

A total of 28 variables determined at hospital admission and associated with a critical illness (Figure 2) were included in the model development. Prediction models were constructed using LASSO logistic regression, SVR, ANN, RT, and MARS, their performance was evaluated by the ROC analysis (Figure 4). Although the predictive ability of ANN and SVR in the development cohort was better than other algorithms, the predictive ability using models of LASSO logistic regression and ANN outperformed the other algorithms in the validating dataset (Figure 4D). The LASSO logistic regression model was selected by us for its high predictive power and interpretability. In LASSO regression, after excluding irrelevant and redundant features (Figures 4A,B), 21 features remained for LASSO regression analysis, including age, whether take ARB drugs and blood test results, lymphocytes, neutrophils, blood platelet, thrombocytocrit, RDW (CV and SD), hematocrit, hemoglobin concentration, AST, CK-MB, albumin, LDH, glucose, K, Na, CRP, PCT, PT, APTT, fibrinogen, and uric acid. The risk score was constructed based on the coefficients from the LASSO logistic model (Table 2) and then converted into a probability with formulas presented in the method and materials section. By internal 100 times bootstrap validation, the mean AUC based on data from the development cohort was 0.83 (95% CI, 0.78–0.86) (Figure 4C). Variables utilized in the risk score for the validation cohort are shown in Table 3. The accuracy of the COVID risk score in the validation cohort was like that of the development cohort with an AUC in the validation cohort of 0.84 (Figure 4D).

**Table 2:** Coefficients of LASSO logistic regression model for predicting development of critical illness in 1,064 patients hospitalized with COVID-19 in the development dataset.

**Laboratory tests**	**Coefficient**
Lymphocytes, ×10^9^/L	−1.0049
Neutrophils, ×10^9^/L	0.085
Blood platelet, ×10^9^/L	0.017
Thrombocytocrit, %	−19.7385
RDW(CV),%	0.0601
RDW(SD), fL	0.0395
Hematokrit, %	−0.003
Hemoglobin concentration, g/L	−0.0015
Glucose, mmol/L	0.1131
K, mmol/L	−0.3833
Na, mmol/L	−0.0187
AST, U/L	−0.0026
CK-MB, U/L	0.0037
Albumin, g/L	0.0096
PT, s	0.1381
APTT, s	0.0148
Fibrinogen, g/L	−0.1319
CRP, mg/L	0.0033
PCT, ng/ml	0.1068
α-HBDH, U/L	0.0005
Uric acid, umol/L	−0.0025

*RDW, red blood cell volume distribution width; AST, aspartate aminotransferase; CV, coefficient of variation; SD, standard deviation; AST, aspartate aminotransferase; CK-MB, Creatine kinase muscle-brain isoform; LDH, lactate dehydrogenase; CRP, C-reactive protein; PCT, Procalcitonin; PT, prothrombin time; APTT, activated partial thromboplastin time*.

**Table 3:** Laboratory characteristics of patients with COVID-19 in validation cohort.

**Laboratory tests**	**Total, mean (SD) [Interquartile range]**	**Critical illness (*n* = 566)**	
		**No (*n* = 538)**	**Yes (*n* = 28)**
**Hematologic**			
Lymphocytes, ×10^9^/L	1.23 (0.57) [0.82–1.55]	1.26 (0.57) [0.86–1.58]	0.78 (0.35) [0.53–1.03]
Neutrophils, ×10^9^/L	3.71 (2.66) [2.19–4.23]	3.61 (2.60) [2.15–4.16]	5.52 (3.11) [2.97–8.07]
Blood platelet, ×10^9^/L	192.7 (74.34) [139–233]	194.46 (74.95) [139–234.25]	144.38 (25.92) [131–154]
Thrombocytocrit, %	0.2 (0.07) [0.15–0.24]	0.2 (0.07) [0.15–0.24]	0.17 (0.05) [0.14–0.2]
RDW(CV),%	12.39 (1.24) [11.8–12.7]	12.37 (1.21) [11.8–12.7]	12.88 (1.63) [11.9–13.45]
RDW(SD), fL	39.65 (3.28) [37.5–41.4]	39.61 (3.25) [37.5–41.4]	40.67 (3.81) [38.1–43.08]
Hematokrit, %	37.39 (10.86) [35.3–42.9]	37.52 (10.9) [35.38–43]	35.05 (10.05) [33.58–39.08]
Hemoglobin concentration, g/L	132.59 (21.4) [122–147]	133.1 (20.97) [122–147]	123.96 (26.73) [119–141]
**Biochemical**			
Glucose, mmol/L	7.19 (3.41) [5.34–7.87]	7.08 (3.26) [5.31–7.7]	9.03 (5.23) [6.26–9.19]
K, mmol/L	3.97 (0.47) [3.64–4.24]	3.98 (0.46) [3.67–4.24]	3.79 (0.65) [3.44–4.13]
Na, mmol/L	138.9 (3.42) [137–140.91]	139.01 (3.42) [137.2–141]	136.61 (2.63) [136–137.8]
AST, U/L	29.75 (15.4) [20–34]	29.06 (14.75) [20–33]	43.06 (21.16) [24.1–54.6]
CK-MB, U/L	13.83 (6.78) [9.99–16.73]	13.67 (6.74) [9.8–16.12]	16.83 (6.87) [13–20.11]
Albumin, g/L	40.81 (5.03) [37.92–44.1]	41.05 (4.93) [38.3–44.4]	36.16 (4.75) [33.5–40.2]
**Infection-related indices**			
CRP, mg/L	22.55 (30.41) [2.9–28.3]	20.48 (28.26) [2.67–26.1]	59.27 (42.61) [25.27–95.5]
PCT, ng/ml	0.08 (0.13) [0.04–0.08]	0.07 (0.09) [0.04–0.08]	0.21 (0.37) [0.04–0.18]
α-HBDH, U/L	200.1 (81.44) [149.68–229.1]	193.16 (76.59) [144.5–221.75]	273.63 (98.73) [203.57–307.15]
**Coagulation function**			
PT, s	11.81 (2.29) [10.7–12.7]	11.69 (1.38) [10.7–12.7]	13.95 (7.69) [11.62–13.2]
APTT, s	31.63 (7.9) [27.55–35.8]	31.28 (7.33) [27.2–35.5]	37.42 (13.41) [31.12–41.9]
Fibrinogen, g/L	6.74 (34.13) [2.93–4.54]	6.89 (35.15) [2.92–4.5]	4.22 (1.24) [3.5–4.99]
Uric acid, umol/L	265.51 (89.47) [202.05–319.18]	267.98 (88.84) [206.1–320.52]	217.79 (89.99) [154.9–254]

*RDW, red blood cell volume distribution width; AST, aspartate aminotransferase; CV, coefficient of variation; SD, standard deviation; AST, aspartate aminotransferase; CK-MB, Creatine kinase muscle-brain isoform; LDH, lactate dehydrogenase; CRP, C-reactive protein; PCT, Procalcitonin; PT, prothrombin time; APTT, activated partial thromboplastin time*.

## Discussion

Early identification of patients with COVID-19 at risk of progression to critical illness disease will aid in better patient management and effective usage of healthcare resources. In this study, we unraveled that older age and higher levels of laboratory test indexes such as CRP, LDH, and glucose, and lower levels of laboratory findings such as lymphocytes and albumin on admission were associated with higher probabilities of critical illness COVID-19. In addition, a clinical risk score based on LASSO logistic regression was developed to predict the development of critical illness patients with COVID-19 with satisfactory accuracy according to AUC (0.83). Generally, the 21 variables required for estimating the probability of developing critical illness can be easily obtained from routine tests at hospital admission. The robustness and applicability of the risk score were confirmed in the independent validation dataset (AUC = 0.84).

Univariate analyses revealed that factors, namely, age, neutrophils, D-dimer, LDH, CRP, glucose, APTT, fibrinogen, AST, and several other biochemical parameters were associated with a critical illness. In addition, the dynamic profile of the significant laboratory findings was tracked. Levels of LDH, D-dimer, glucose, CRP, α-HBDH, and globulin are higher in the critical illness group compared with the non-critical illness group. And neutrophil counts and albumin are lower in the critical illness group compared with the non-critical illness group. A prediction model for critical illness was developed with 21 predictors that were found to be independently correlated with critical illness *via* multivariate LASSO logistic regression analysis. Previous studies have found several of these variables to be prognosis factors for patients with COVID-19. It has been reported that elderly patients were more commonly critically ill with COVID-19 (3, 26, 27) and have a higher probability of a death outcome (28, 29). Modelli and colleagues revealed that the 28-day fatality rate was associated with increasing age, hypertension, cardiovascular disease, and higher body mass index (17), in agreement with the previous work.

Lymphopenia, leukocytosis (with increased absolute neutrophil counts), eosinopenia, neutrophilia, increased CRP and PCT which reflects a persistent state of inflammation (30) may be related to cytokine storm and cellar immune deficiency induced by virus invasion (27, 31). Zhou et al. found lower lymphocyte counts and higher LDH in patients who died from COVID-19 (1). Injured alveolar epithelial cells could lead to the infiltration of lymphocytes, resulting in persistent lymphopenia (32, 33). Lymphopenia is a common characteristic in patients with COVID-19 and might play an important role in the disease process (34, 35). Zhang et al. noted that 53% of patients admitted with COVID-19 had eosinopenia on the day of hospital admission (36). Calabrese et al. reported that lymphocyte and platelet counts were the most important features able to stratify patients into different clinical clusters (37). Ewan et al. demonstrated that risk stratification was improved by blood and physiological parameters (C-reactive protein, neutrophil/lymphocyte ratio, and neutrophil count) measured at hospital admission (20). Such findings were consistent with this work. A higher level of LDH was an indication of the activity and severity of idiopathic pulmonary fibrosis and is one of the most important prognostic biomarkers of lung injury (37). LDH was reported to be higher in severe and patients who received ICU treatment with COVID-19 than in mild and non-ICU patients (27, 30, 38, 39), which is utilized as a valuable prognosis predictor (40, 41). In addition, patients with elevated CK-MB levels on hospital admission were at significantly increased risk of critical illness. Li and colleagues found that cardiac injury (elevated LDH and CK-MB levels) were associated with severe disease or ICU admission and death in patients with COVID-19 (42). Increased PT and APTT, decreased blood platelet, thrombocytocrit, and fibrinogen which reflect the coagulation activation might be associated with the sustained inflammatory response. Banoei et al. noted that prothrombin and lactate were the most differentiating biochemical markers in the mortality prediction model (18).

Since hyperglycemia is harmful to the management of inflammation and viremia, the association between the level of glucose and critical illness in COVID-19 viral infections is not surprising. Based on big data analysis with a cohort with 7,337 COVID-19 cases, Zhu et al. revealed that diabetics with better-controlled blood glucose were associated with a decreased death risk than diabetics with poorly controlled blood glucose (43). Banoei and colleagues demonstrated that disease, coronary artery disease, dementia, age > 65, and altered mental status were the topmost differentiating mortality predictors (22).

Previous studies have identified that 15–53% of cases reported abnormal levels of AST during disease progression (44–47). In a study conducted by Huang et al. (48), the elevation of AST was found in 8 (62%) of 13 patients in the ICU compared with 7 (25%) of 28 COVID-19 infected cases who did not need ICU care. Abnormal liver tests occur in most hospitalized patients with COVID-19 and may be associated with ICU admission, mechanical ventilation (48), and death (28, 48). Liver damage (decreased albumin and increased globulin) in patients with COVID-19 infections might be associated with the direct effect of the viral infection of liver cells, drug hepatotoxicity, or immune-mediated inflammation (37), such as cytokine storm and pneumonia-associated hypoxia.

Prediction models for the dynamic and control of COVID-19 infection found broad similarities with the features retained in our models, particularly regarding aging, hypertension, CRP, LDH, prothrombin, lactate, and neutrophil levels (14–24). The main advantage of the LASSO logistic regression is that the variable with a large parameter estimation is compressed to a smaller variable, while the variable with the smaller parameter estimate is compressed to 0. The parameter estimation of the LASSO analysis is continuous, which is suitable for model selection with high-dimensional data.

In the development dataset, we found that the discriminative abilities of SVR, ANN, RT, and MARS were outperforming that of LASSO logistic regression as evaluated by AUCs. However, in the independent validation dataset, the predictive ability of LASSO logistic regression was the best within all algorithms and was selected by us. The phenomenon that the model that incorporates the highest level of non-linearity displayed better in-sample prediction, but also yielded the worse out-of-sample performances may account for the over-fitting problem of the ANN, RT, MARS, and SVR algorithms (45). The linear Kernel function utilized in LASSO logistic regression performed badly in-sample but generated the best out-of-sample predictions.

There are inevitably limitations in our retrospective study. The primary one is incomplete laboratory findings in the electronic database and the lacking of CT images, which decreases the statistical power of the LASSO logistic regression model. Therefore, important information might be missed and further prospective studies are required. However, our model has a certain tolerance to missing data, as high performance as measured by AUC on the developing and external validation dataset for samples missing 20% of the predictors was achieved. Second, since the algorithms we tried are purely data-driven, the performances of these models may vary if developed with different datasets. We believe that more accurate models can be obtained with the increasing of available datasets. Third, the data for risk probability development and validation are from two provinces of China, which could potentially limit the generalizability of the risk model. Further studies on different populations all over the world with larger patient cohorts are needed to validate our findings.

## Conclusion

In summary, this study identified 28 indicators (such as age, LDH, CRP, and lymphocytes) associated with critical illness of patients with COVID-19. The longitudinal laboratory variables were explored. A risk score to estimate the risk of developing critical illness among patients with COVID-19 was developed based on 21 variables independently associated with critical illness and commonly measured on hospital admission. The risk model is especially valuable for early detection and intervention of the incidence of critical illness COVID-19, thus making improvements to clinical strategies against COVID-19, optimizing the use of healthcare resources, and potentially reducing mortality in patients with COVID-19.

## Data Availability Statement

The raw data supporting the conclusions of this article will be made available by the authors, without undue reservation.

## Author Contributions

YF: conceptualization and writing. WZho: resources and data curation. TL, JL, KX, XM, LX, and JJ: resources. HZ: supervision. RL: project administration and supervision. WZha: funding acquisition. All authors contributed to the article and approved the submitted version.

## Funding

This study was supported by the National Scientific Foundation of China (Nos. 81874329, 81573511, and 81522048).

## Conflict of Interest

YF was employed by Cofoe Medical Technology Co., Ltd. The remaining authors declare that the research was conducted in the absence of any commercial or financial relationships that could be construed as a potential conflict of interest.

## Publisher's Note

All claims expressed in this article are solely those of the authors and do not necessarily represent those of their affiliated organizations, or those of the publisher, the editors and the reviewers. Any product that may be evaluated in this article, or claim that may be made by its manufacturer, is not guaranteed or endorsed by the publisher.
