# Supplementary material for: Early Prediction Model for Critical Illness of Hospitalized COVID-19 Patients Based on Machine Learning Techniques
Source: Front Public Health. 2022 May 24;10:880999. doi: 10.3389/fpubh.2022.880999 (PMC9168534; doi:10.3389/fpubh.2022.880999)
Supplement: Supplementary file 1 [file Table_1.DOCX]

**Supplementary**

**e-Table 1. The laboratory findings and corresponding missing ratio in the development cohort.**

| **type** | **Test name** | **Abbreviations** | **Unit** | **Ratio of missing value** |
| --- | --- | --- | --- | --- |
| Hematologic | Hematokrit |  | % | 3.90% |
| Hematologic | Basophils |  | 10^9/L | 4.06% |
| Hematologic | Eosnophils |  | 10^9/L | 4.06% |
| Hematologic | Hemoglobin concentration |  | g/L | 4.06% |
| Hematologic | Mean corpuscular volume | MCV | fL | 4.06% |
| Hematologic | Monocytes |  | 10^9/L | 4.06% |
| Hematologic | Neutrophils |  | 10^9/L | 4.06% |
| Hematologic | Red blood cell volume distribution width_coefficient of variation | RDW (CV) | % | 4.06% |
| Hematologic | Red blood cell volume distribution width_standard deviation | RDW (SD) | fL | 4.14% |
| Hematologic | Lymphocytes |  | 10^9/L | 4.30% |
| blood chemistry | Glucose |  | mmol/L | 5.90% |
| blood chemistry | K |  | mmol/L | 6.06% |
| blood chemistry | Na |  | mmol/L | 6.06% |
| Hematologic | Blood platelet count |  | 10^9/L | 6.14% |
| liver function | Aspartate aminotransferase | AST | U/L | 6.14% |
| others | Uric acid |  | umol/L | 6.22% |
| renal function | Creatinine | Cr | μmol/L | 6.22% |
| renal function | Ureophil |  | mmol/L | 6.29% |
| Hematologic | Thrombocytocrit |  | % | 6.77% |
| blood chemistry | Total Ca |  | mmol/L | 6.85% |
| blood chemistry | Cl |  | mmol/L | 6.93% |
| blood chemistry | Total protein |  | g/L | 7.25% |
| liver function | Glutamic-pyruvic transaminase |  | U/L | 7.25% |
| renal function | Albumin |  | g/L | 7.25% |
| renal function | Albumin to globulin ratio |  | % | 7.49% |
| renal function | Globulin |  | g/L | 7.49% |
| Hematologic | Red blood cell |  | 10^12/L | 8.37% |
| Hematologic | White blood cells |  | 10^9/L | 13.07% |
| Infection-related indices | C-reactive protein | CRP | mg/L | 14.82% |
| Coagulation function | D dimer |  | μg/Ml | 22.87% |
| Coagulation function | Activated partial thromboplastin time | APTT | Sec | 23.27% |
| Coagulation function | Fibrinogen |  | g/L | 23.27% |
| Coagulation function | Prothrombin time | PT | Sec | 23.27% |
| Infection-related indices | Procalcitonin | PCT | ng/ml | 33.47% |
| blood chemistry | Lactic dehydrogenase | LDH | U/L | 34.34% |
| liver function | Creatine kinase |  | U/L | 34.34% |
| liver function | Creatine kinase muscle-brain isoform | CK-MB | U/L | 40.72% |
| Infection-related indices | alpha hydroxybutyrate dehydrogenase | α-HBDH | U/L | 42.71% |
| renal function | Glomerular filtration rate | GFR | ml/min | 52.27% |
| liver function | Alkaline phosphatase |  | U/L | 54.50% |
| liver function | Prealbumin |  | mg/L | 54.90% |
| liver function | Total bile acid |  | μmol/L | 54.90% |
| Coagulation function | Prothrombin time activity |  | % | 57.29% |
| Coagulation function | Prothrombin time ratio |  |  | 57.29% |
| Infection-related indices | Erythrocyte sedimentation rate | ESR | mm/h | 60.32% |
| renal function | Cystatin C |  | mg/L | 61.91% |
| lipid profile | Total cholesterol |  | mmol/L | 65.18% |
| lipid profile | High-density lipoprotein | HDL | μmol/L | 65.26% |
| lipid profile | Low density lipid cholesterol | LDL | mmol/L | 65.26% |
| lipid profile | Triglyceride |  | mmol/L | 65.26% |
| Coagulation function | international normalized ratio |  |  | 65.98% |
| blood gas assay | Partial pressure of carbon dioxide |  | mmHg | 67.73% |
| blood gas assay | pO2 |  | mmHg | 67.73% |
| blood gas assay | sO2 |  | % | 67.73% |
| blood chemistry | Base excess |  | mmol/L | 67.81% |
| others | Retinol conjugated protein |  | mg/L | 69.24% |
| lipid profile | Apolipoprotein A1 |  | g/L | 69.72% |
| lipid profile | Apolipoprotein B |  | g/L | 69.72% |
| liver function | Alpha-l fucosidase |  | U/L | 71.16% |
| liver function | Monoamine oxidase |  | U/L | 71.16% |
| blood chemistry | Angiotensin converting enzyme |  | U/L | 71.16% |
| lipid profile | Free fatty acid |  | mmol/L | 73.07% |
| blood chemistry | Type B natriuretic peptide |  | pg/ml | 74.18% |
| blood chemistry | Ischemia Modified Albumin |  | U/mL | 75.30% |
| liver function | Homocysteine |  | μmol/L | 76.25% |
| others | urine specific gravity |  | NA | 76.33% |
| others | Sialic acid |  | mg/dL | 77.13% |
| blood chemistry | ceruloplasmin |  | mg/dL | 77.13% |
| blood chemistry | Ca2+ |  | mmol/L | 79.84% |
| blood chemistry | Residual base in extracellular fluid |  | mmol/L | 79.84% |
| blood chemistry | Hypersensitive troponin I |  | ng/mL | 79.92% |
| blood chemistry | Precursor of Type B natriuretic peptide |  | pg/mL | 80.16% |
| blood chemistry | cholinesterase |  | U/L | 80.24% |
| blood chemistry | troponin |  | μg/L | 80.80% |
| blood chemistry | Mg |  | mmol/L | 81.04% |
| blood chemistry | myohemoglobin |  | ng/ml | 84.46% |
| lipid profile | lipase |  | U/L | 86.85% |
| blood chemistry | Standard base surplus |  | mmol/L | 87.89% |
| blood gas assay | Bicarbonate ions |  | mmol/L | 87.89% |
| blood chemistry | Alpha 1 microglobulin |  | mg/L | 87.97% |
| lipid profile | Lipoprotein α |  | mg/L | 91.00% |
